# Early Recovery of *Salmonella* from Food Using a 6-Hour Non-selective Pre-enrichment and Reformulation of Tetrathionate Broth

**DOI:** 10.3389/fmicb.2016.02103

**Published:** 2016-12-27

**Authors:** Ninalynn Daquigan, Christopher J. Grim, James R. White, Darcy E. Hanes, Karen G. Jarvis

**Affiliations:** ^1^Office of Applied Research and Safety Assessment, Center for Food Safety and Applied Nutrition, U.S. Food and Drug AdministrationLaurel, MD, USA; ^2^Oak Ridge Institute for Science and TechnologyOak Ridge, TN, USA; ^3^Resphera Biosciences Baltimore, MD, USA

**Keywords:** *Salmonella*, FDA BAM, metagenomics, 16S rRNA, selective enrichment, tetrathionate broth

## Abstract

Culture based methods are commonly employed to detect pathogens in food and environmental samples. These methods are time consuming and complex, requiring multiple non-selective and selective enrichment broths, and usually take at least 1 week to recover and identify pathogens. Improving pathogen detection in foods is a primary goal for regulatory agencies and industry. *Salmonella* detection in food relies on a series of culture steps in broth formulations optimized to resuscitate *Salmonella* and reduce the abundance of competitive bacteria. Examples of non-selective pre-enrichment broths used to isolate *Salmonella* from food include Lactose, Universal Pre-enrichment, BPW, and Trypticase Soy broths. Tetrathionate (TT) and Rappaport–Vassiliadis (RV) broths are employed after a 24-h non-selective enrichment to select for *Salmonella* and hamper the growth of competitive bacteria. In this study, we tested a new formulation of TT broth that lacks brilliant green dye and has lower levels of TT . We employed this TT broth formulation in conjunction with a 6-h non-selective pre-enrichment period and determined that *Salmonella* recovery was possible one day earlier than standard food culture methods. We tested the shortened culture method in different non-selective enrichment broths, enumerated *Salmonella* in the non-selective enrichments, and used 16S rRNA gene sequencing to determine the proportional abundances of *Salmonella* in the TT and RV selective enrichments. Together these data revealed that a 6-h non-selective pre-enrichment reduces the levels of competitive bacteria inoculated into the selective TT and RV broths, enabling the recovery of *Salmonella* 1 day earlier than standard culture enrichment methods.

## Introduction

The impact of *Salmonella* food contamination on public health has resulted in the development of several comprehensive culture-based methods to detect *Salmonella* in food. These are found namely in the Food and Drug Administration (FDA) Bacteriological Analytical Manual (BAM), the United States Department of Agriculture (USDA) Microbiological Laboratory Guidebook (MLG), and the ISO 6579:2002 Microbiology of food and animal feeding stuffs- Horizontal method for the detection of *Salmonella* species (ISO) (ISO, 2002; USDA, 2014; FDA, 2015). The current method used by the FDA, described in the BAM, requires a 24-h resuscitation in non-selective pre-enrichment broth, followed by parallel 24-h selective enrichments in Tetrathionate (TT) and Rappaport–Vassiliadis (RV) broths to reduce the growth of competitive bacteria (FDA, 2015). Aliquots of TT and RV enrichments are then plated on selective agars, which are incubated for 24 to 48-h. Presumptive *Salmonella* colonies are isolated for confirmatory testing which can take an additional 2 to 3 days.

Important considerations for successful recovery of *Salmonella* from food include the type of food, the level of stress or injury imparted on the *Salmonella* by the food matrix or processing environment, the presence of competitive bacteria, and the level of *Salmonella* contamination (D’Aoust, 1981; Busse, 1995). Previous studies noted the importance of resuscitating stressed and injured *Salmonella*, so current methods utilize specific non-selective pre-enrichment broths to recover *Salmonella* from food (Rappaport et al., 1956; D’Aoust and Maishment, 1979; D’Aoust, 1981; Ray, 1989; Chen et al., 2013). Supplements such as bile salts, brilliant green dye, MgCl_2_, and malachite green dye are added to broths used for selective enrichment to reduce competitive bacteria in foods tested for *Salmonella* contamination (Teague and Clurman, 1916; Rappaport et al., 1956; Vassiliadis et al., 1978; Peterz et al., 1989). For example, the low levels of *Salmonella*, enumerated from pine nuts (0.028–0.093 MPN/g) and paprika (0.04–0.05 CFU/g) implicated in recent outbreaks, highlight the need for non-selective pre-enrichment prior to selective enrichment to increase the probability of detection, especially for injured or stressed organisms that would not survive in selective enrichment broths (Lehmacher et al., 1995; Wang et al., 2015).

Common to all of the detection methods employed for the recovery of *Salmonella* from food, regardless of the type of food or non-selective pre-enrichment broth, is the use of TT and RV selective broths. The selective action of TT includes: bile salts and brilliant green dye to inhibit gram-positive bacteria; an iodine–iodide (I_2_–KI) solution added to the base broth to induce TT production, providing a metabolic advantage to organisms that have TT reductase such as *Salmonella*; and calcium carbonate to neutralize the sulfuric acid produced when TT is reduced, thus maintaining the pH (Teague and Clurman, 1916; Knox, 1945; Palumbo and Alford, 1970; Moats et al., 1974; D’Aoust, 1981; Moats, 1981). *Salmonella* growth in TT broth relies on the ability of *Salmonella* to reduce TT and withstand the bactericidal activity of brilliant green dye. It is notable that during infection in the human intestine *Salmonella* induces inflammation of gut tissues to induce TT production which it can use as an energy source through anaerobic respiration, giving this organism a competitive advantage over competing bacteria that use fermentation in this anaerobic environment (Winter et al., 2010).

Rappaport–Vassiliadis broth has different selective properties to improve *Salmonella* recovery, namely MgCl_2_ at a concentration that creates hypertonic conditions that inhibit *Proteus* and *Escherichia coli*, and malachite green to inhibit other competing bacteria (Rappaport et al., 1956; Vassiliadis et al., 1978; Peterz et al., 1989). It is important to use MgCl_2_ instead of other Mg salts, such as MgS_2_0_3_, MgBr_2_, Mg(N0_3_)_2_, BaCl_2_, CaS_2_0_3_.Ca(N0_3_)_2_, CaBr_2_, Cal_2_, KNO_3_, or BeCI_2_ in RV, as these other salts are not as selective (Rappaport et al., 1956). The low pH of RV, well tolerated by *Salmonella*, provides additional selection against coliforms (Rappaport et al., 1956; Vassiliadis et al., 1981; Vassiliadis, 1983). Together the attributes of RV broth, MgCl_2_, low pH, and malachite green, provide a high osmotic pressure, a low pH that declines over time with bacterial metabolism, and inhibition of gram-positive organisms, respectively, that provide favorable conditions for *Salmonella* growth (van Schothorst and Renaud, 1985; Peterz et al., 1989). An additional selective feature of both RV and TT broths are incubation temperatures above 37°C to reduce competitive bacteria (D’Aoust, 1981; Peterz et al., 1989). The FDA BAM recommends incubation of RV medium for 24 ± 2 h at 42 ± 0.2°C and incubation of TT broth for 24 ± 2 h at 43 ± 0.2°C (FDA, 2015).

Despite the efficacy and proven success of current culture-based methods for *Salmonella* detection, reducing detection time is a priority for food safety. Approaches to reduced detection time include reducing the length of non-selective and selective enrichments, changing the broth formulations, and altering incubation temperatures. Attempts to alter enrichment times and temperatures have met with mixed results (Mohr et al., 1974; van Schothorst and van Leusden, 1975; D’Aoust and Maishment, 1979). One investigation determined *Salmonella* was able to resuscitate sufficiently after 5 to 6-h in a non-selective pre-enrichment broth and could overcome the toxic effects of selective enrichment (Chen et al., 1993). However, most early studies with shortened non-selective pre-enrichments were unsuccessful (Mohr et al., 1974; van Schothorst and van Leusden, 1975; D’Aoust and Maishment, 1979). Additional studies to shorten the time for selective enrichment also failed due to false negative results, especially in low moisture foods (D’Aoust et al., 1990).

The choice of non-selective and selective pre-enrichment broths depends on the food or environmental samples being tested. The FDA BAM, USDA MLG, and ISO manuals specify enrichment media formulations that are suitable for *Salmonella* detection in foods based on characteristics such as pH, high versus low microbial loads in the food, and the moisture content of the food (ISO, 2002; USDA, 2014; FDA, 2015). Foods that are regulated by the FDA are divided into 35 categories that require 15 different pre-enrichment broths (FDA, 2015).

Molecular methods, such as qPCR, and automated platforms, such as the VIDAS Easy, have successfully detected *Salmonella* in 24-h non-selective pre-enrichments of pine nuts, chili powder, soft cheese, raw fish, and tomatoes, thus reducing the detection time (Cheng et al., 2009; Wang et al., 2015). However, culture based methods are considered the gold standard for regulatory agencies highlighting the importance of reducing the culture-dependent detection time. Methods such as Pulse Field Gel Electrophoresis and whole genome sequencing currently employed for traceback analysis during foodborne outbreak investigations require pure cultures of bacteria (Swaminathan et al., 2001; Allard et al., 2016).

Recent advances in high-throughput DNA sequencing provide opportunities to profile commodity-associated microbiomes either through amplicon sequencing of the 16S rRNA gene or whole-genome shotgun metagenomic sequencing. Metagenomic sequencing provides accurate data about the entire microbial community including organisms that cannot be cultured using traditional methods (Caporaso et al., 2012). Studies aimed at improving foodborne pathogen detection have utilized microbiome profiling to characterize food microbiomes and follow microbial community shifts throughout the BAM culture process (Pettengill et al., 2012; Ottesen et al., 2013; Jarvis et al., 2015; Leonard et al., 2015). These studies also demonstrate the value of microbiome profiling for characterizing the growth of *Salmonella* and *Escherichia coli* pathogens amidst the complex bacterial communities that naturally inhabit leafy green produce and tomatoes (Pettengill et al., 2012; Ottesen et al., 2013; Jarvis et al., 2015; Leonard et al., 2015). The approach is particularly suitable for characterizing competitive members of the microbial community that will hamper detection of pathogens in contaminated foods.

In this study, we employed a combination of microbiology and metagenomic methods to characterize and test potential changes to the FDA BAM method for *Salmonella* detection in leafy greens. First, four non-selective pre-enrichment broths including Lactose broth, Modified Buffered Peptone Water (mBPW), Trypticase Soy Broth (TSB), and Universal Pre-enrichment broth (UP), were compared in the FDA BAM culture method using cilantro as a model for leafy green produce. Second, we tested changes in the FDA BAM TT broth formulation that reduced selective pressure by eliminating brilliant green and reducing the concentration of iodine as follows: (1) TT, formulated according to the FDA BAM with brilliant green and 2% I_2_-KI solution; (2) TT_A_, no brilliant green, 2% I_2_-KI solution; and (3) TT_B_, no brilliant green, 1% I_2_–KI solution. Third, we tested the efficacy of 5 and 6-h non-selective pre-enrichment times in conjunction with the different TT broth formulations, for *Salmonella* recovery from food.

We evaluated the efficacy of these changes to the BAM method by enumerating *Salmonella* contamination levels in the non-selective pre-enrichments and employed 16S rRNA gene amplicon sequencing to define the microbial communities in selective TT and RV broth enrichments. Finally, we tested the combination of a reduced non-selective pre-enrichment time and reduced TT selectivity for *Salmonella* recovery from raw chicken thighs, liquid whole eggs, and peanut butter.

This study demonstrates that a less selective formulation of the BAM TT broth, inoculated with a 6-h non-selective pre-enrichment, consistently recovered *Salmonella* one day earlier than the current FDA BAM culture method. Additionally, we show that this method can be adapted for use with other food commodities, which is advantageous for Public Health Laboratories that test a variety of foods.

## Materials and Methods

### Foods Matrices Tested in This Study

Four foods were tested in this study including cilantro, raw chicken thighs, liquid whole eggs, and peanut butter. Cilantro samples, provided by the Department of Agriculture and Rural Development in Lansing, Michigan or purchased from a local grocery store, were stored at 4°C prior to testing. Raw chicken thighs and liquid whole eggs, purchased from a local supermarket, were stored at 4°C prior to testing. Peanut butter samples, purchased from a local grocery store, were stored at room temperature.

### Bacterial Strains for Food Inoculations

Nine *Salmonella enterica* strains representing four serovars, Newport, Tennessee, Thompson, and Enteritidis, were used for food inoculation (**Table 1**). Two of the strains used to inoculate cilantro, *S*. Newport SALC14 and *S*. Tennessee SALC 76, were cultured from cilantro in our laboratory and the third strain, *S*. Thompson SALC 818, was chosen because *S*. Thompson was implicated in a cilantro outbreak in 1999 (Campbell et al., 2001). The three *S*. Tennessee strains used to inoculated peanut butter were isolated from peanut butter during a 2007 peanut butter outbreak (Wilson et al., 2016) and the *S*. Enteritidis strains used for the chicken and egg inoculations were isolated from the respective foods (**Table 1**). Culture stocks, stored at –80°C, were streaked onto Trypticase Soy Agar plates (Difco, Sparks, MD, USA) and incubated overnight at 35 ± 2°C. Bacterial cell suspensions, equal to 0.48–0.52 McFarland turbidity units (corresponding to approximately 1 × 10^8^ CFU/ml), were prepared in 0.85% sterile saline and serially diluted to approximately 28 CFU/mL for food inoculations.

**Table 1:** *Salmonella* strains used to inoculate foods.

DVA name	Nickname	Serotype	Origin	State/Country	Food inoculated	Non-selective pre-enrichment broths tested
SALC 14	N/A	Newport	Cilantro	New York, USA	Cilantro	Lactose, mBPW
SALC 76	N/A	Tennessee	Cilantro	New York, USA	Cilantro	mBPW, TSB, UP
SAL 818	TXAML1201424	Thompson	N/A	Texas, USA	Cilantro	mBPW, TSB, UP
SAL 622	SL487	Tennessee	Peanut Butter Outbreak	Minnesota, USA	Peanut Butter	mBPW
SAL 623	SL488	Tennessee	Peanut Butter Outbreak	Minnesota, USA	Peanut Butter	mBPW
SAL 624	SL489	Tennessee	Peanut Butter Outbreak	Tennessee, USA	Peanut Butter	mBPW
SAL 274	B7849	Enteritidis	Chicken	Spain	Liquid whole eggs	TSB with FeSO_4_
SAL 274	B7849	Enteritidis	Chicken	Spain	Raw Chicken thighs	BPW
SAL 311	SL915	Enteritidis	Egg Yolk	N/A	Liquid whole eggs	TSB with FeSO_4_
SAL 311	SL915	Enteritidis	Egg Yolk	N/A	Raw Chicken thighs	BPW
SAL 336	SL940	Enteritidis	Egg	N/A	Liquid whole eggs	TSB with FeSO_4_
SAL 336	SL940	Enteritidis	Egg	N/A	Raw Chicken thighs	BPW

Prior to inoculation, liquid whole eggs were homogenized in sterile beakers, and the raw chicken thighs were cubed into approximately 1-inch squares using sterile knives. All foods were aseptically portioned (25 g) into sterile Whirlpak bags (Nasco; Fort Atkinson, WI, USA), inoculated with approximately 28 CFU *Salmonella* per 25 g of food, and aged to simulate natural contamination at 4°C for 48–72 h (cilantro, liquid whole eggs, and chicken thighs) or at room temperature for 14 days (peanut butter). Un-inoculated controls samples were also prepared and aged in parallel for each food.

### Non-selective Pre-enrichment of Foods

Aged cilantro samples were enriched in Lactose broth, UP or TSB prepared according to the FDA BAM. Peanut butter and additional cilantro samples were enriched in Modified Buffer Peptone Water [mBPW; Buffered Peptone Water (BPW, Difco^TM^, Sparks, MD, USA), with 3.5 g of disodium phosphate and 1.5 g of monopotassium phosphate per liter]. Aged liquid whole eggs were enriched in TSB with ferrous sulfate prepared according to the FDA BAM. The aged chicken thighs were enriched in BPW, the non-selective pre-enrichment broth recommended in the MLG method for the isolation and identification of *Salmonella* from poultry.

### Selective Enrichment Broths

Two modifications that reduce the selective strength of TT broth were compared to the standard TT broth formulation used in the FDA BAM. Standard BAM TT broth consists of a base broth to which Iodine-Potassium Iodide (I_2_-KI) and brilliant green dye solutions are added on the day of use. Both TT modifications used in this study consist of the TT broth base without the addition of brilliant green dye. TT modification A (TT_A_) consists of the TT broth base with 2.0% of the I_2_–KI solution as per the BAM, and TT modification B (TT_B_) contains only 1.0% of the I_2_–KI solution. The FDA BAM, USDA MLG, and ISO methods all use a second selective enrichment broth called RV for *Salmonella* detection. The RV used in this study was prepared in accordance with the FDA BAM method.

A panel of 101 *Salmonella* isolates, derived from the Defense Science Office (DSO) of the Defense Advanced Research Projects Agency (DARPA), Systems and Assays for Food Examination (SAFE) Program, were cultured in selective RV, TT, TT_A_, and TT_B_ broths to compare efficacy for *Salmonella* recovery. The SAFE collection is used at the FDA for validating new methods for *Salmonella* detection in food. The collection is divided into three groups representing a *Salmonella* subspecies set (55 strains), an outbreak cluster set (26 strains), and a food set (20 strains, Supplementary Table S1). Parallel sets of RV, TT, TT_A_, and TT_B_ selective broths were inoculated with 10^3^ or 10^5^ CFU of each *Salmonella* isolate and incubated at 42 ± 2°C (RV) or 43 ± 0.2°C (TT, TT_A_, and TT_B_) for 24 ± 2-h. The following day 10 μl of each culture was plated onto Xylose-Lysine-Desoxycholate Agar (XLD, Becton, Dickinson and Company, Sparks, MD, USA) agar, and after a 24 h incubation at 35 ± 2°C, the plates were recorded as positive (black colonies) or negative (no growth) for *Salmonella*.

### 24-hour Non-selective Pre-enrichment with TT Modifications

**FIGURE 1:**
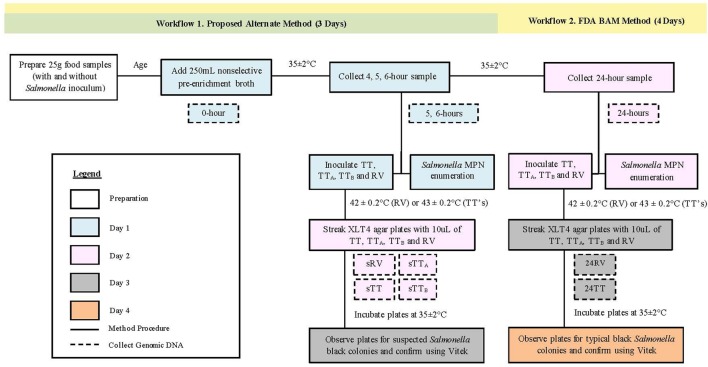
**Workflow for food processing and sample collection.**All experiments used *Salmonella* positive and negative food samples that were aged and processed for *Salmonella* detection using two workflows. Non-selective enrichment samples were collected at 5, and 6-h (workflow 1, green) and 24-h (workflow 2, yellow) for *Salmonella* enumeration, inoculation of selective TT and RV enrichments and 16S rRNA gene sequencing. Daily sample processing steps were performed as outline on Day 1 (Blue), Day 2 (Pink), Day 3 (Gray), and Day 4 (Orange).

All food samples were processed following the FDA BAM workflow for the detection of *Salmonella* with a 24-h non-selective pre-enrichment and the standard and modified TT broths, as follows. On day one after aging, food samples were aseptically combined with 250mL of sterile non-selective pre-enrichment broth, massaged for 2 min, and incubated overnight at 35 ± 2°C (**Figure 1**). On day two, aliquots of the 24-h non-selective pre-enrichments were aseptically transferred to RV (100 μL), TT (1.0 mL), TT_A_ (1.0 mL), and TT_B_ (1.0 mL) selective enrichment broths, and incubated overnight at 42 ± 0.2°C (RV) or 43 ± 0.2°C (TT) (**Figure 1**). On day three, 10 μL of each selective enrichment was plated on Xylose Lysine Tergitol^TM^ 4 (XLT4, Becton, Dickinson and Company, Sparks, MD, USA) agar and incubated overnight at 35 ± 2°C. The remaining volumes of RV, TT, TT_A_, and TT_B_ selective enrichments from cilantro samples were centrifuged at 7,100 rcf for 30 min to pellet the bacteria for DNA extraction, to represent 24RV, 24TT, 24TT_A_, and 24TT_B_ time points used for 16S rRNA gene sequencing (**Figure 1**). Bacterial pellets were stored at –20°C. On day four, suspect black colonies from XLT4 plates were confirmed as *Salmonella* on the VITEK 2 system (BioMérieux, France).

### Reduced Incubation Time for Non-selective Pre-enrichments

In addition to the samples processed according the FDA BAM timeline, replicate samples of each food were incubated for a reduced time in their respective pre-enrichment broths (**Figure 1**). Specifically, on day one, the non-selective pre-enrichments were incubated at 35 ± 2°C for 5 or 6-h, and aliquots from each time period were transferred to RV (100 μL), TT (1.0 mL), TT_A_ (1.0 mL), and TT_B_ (1.0 mL) selective broths and incubated overnight at 42 ± 0.2°C (RV) or 43 ± 0.2°C (TT). On day two, each selective broth was streaked onto XLT4 agar and incubated overnight. Remaining volumes of RV, TT, TT_A_, and TT_B_ selective broths from each time point were centrifuged at 7,100 rcf for 30 min to pellet the bacteria for DNA extraction to represent 5 and 6-h RV, TT, TT_A_, TT_B_ samples (hereafter referred to as sRV, sTT, sTT_A_, and sTT_B_) for 16S rRNA gene sequencing (**Figure 1**). All bacterial pellets were stored at –20°C. On day three, one day earlier than the FDA BAM method, suspect black colonies from XLT4 agar plates were confirmed as *Salmonella* on the VITEK 2 system.

### Enumeration of *Salmonella* in Cilantro

Cilantro non-selective pre-enrichments incubated for 5, 6, and 24-h were enumerated for *Salmonella* using the Most Probable Number (MPN) method described in the FDA BAM. Non-selective pre-enrichment samples were serially diluted (10-fold) in 0.85% NaCl. Selective TT_B_ was inoculated, in triplicate, with 1.0 mL of each dilution, incubated at 43 ± 0.2°C for 48-h, and then streaked onto XLT4. The XLT4 agar plates were examined for the presence of typical black *Salmonella* colonies, which were confirmed in the VITEK 2 system. The FDA BAM MPN calculator was used to compute the results (www.fda.gov/Food/FoodScienceResearch/LaboratoryMethods/ucm109656.htm).

### Preparation of Genomic DNA

Bacterial pellets from the selective RV, TT, TT_A_, and TT_B_ enrichment samples were lysed for 20-min at room temperature with 100 ng/uL lysozyme (Sigma–Aldrich; Saint Louis, MO, USA), followed by DNA extraction on the QIACube using the QIAamp DNA mini protocol (QIAGEN, Germany).

### 16S rRNA Gene Amplification of Selective Enrichments

A two-step PCR amplification strategy was used to generate barcoded 16S rRNA gene amplicons with four sets of PCR primers targeting the Variable 1 to Variable 3 (V1 to V3) regions of the gene. These primers have added base pairs upstream of the 16S rRNA gene aimed at adding nucleotide base diversity to the initial sequencing cycles on the Illumina MiSeq (Supplementary Table S2). Four master mixes were prepared, one for each primer set, consisting of 3.0 ng template DNA, 5.0 μl 5× Omni Klentaq Master Mix (DNA Polymerase Technology, Inc., St. Louis, MO, USA), 10.0 μl PCR Enhancer Cocktail (DNA Polymerase Technology, Inc., St. Louis, MO, USA), and 1.5 μl each forward and reverse primers (600nM final concentration). PCR cycling conditions consisted of a 2-minute denaturation at 94°C, followed by 25 cycles of 40 s at 94°C, 15 s at 56°C, 40 s at 68°C, and a final extension at 68°C for 5 min. The 16S rRNA gene PCR amplicons were visualized on 1% agarose gels and, purified using the QIAquick PCR purification kit (QIAGEN, Germantown, MD, USA) if multiple bands were present.

The second set of PCR reactions used the 16S rRNA gene amplicon PCR products as template with the Illumina Nextera XT indexing primers to generate unique PCR amplicons that were compatible with the Illumina MiSeq sequencing chemistry. Briefly, the 16S rRNA gene amplicons were quantified using the Qubit 2.0 Fluorometer (Life Technologies, Carlsbad, CA, USA), then amplified in 50 μl reactions with ∼150 ng amplicon products, 5× Omni Klentaq Master Mix, and 5 μl each of the Illumina i7 and i5 indexing primers from the Nextera XT Indexing Kit (Illumina Inc., San Diego, CA, USA). Indexing PCR cycling conditions included initial 3 min and 30 s holds at 72 and 98°C, respectively, followed by 5 cycles of 10 s at 98°C, 30 s at 63°C, and 3 min at 72°C, and a final hold at 10°C.

### Library Preparation and 16S rRNA Gene Sequencing

Libraries were size selected using Agencourt AMPure XP beads (Beckman Coulter, Brea, CA, USA) to an average library size of 600 bp, and then quantified using the Qubit 2.0 Fluorometer. Library quality was verified on the Agilent Technologies 2100 Bioanalyzer (Agilent Technologies, Inc., Santa Clara, CA, USA) using the DNA 1000 chip kit. Size selected libraries were normalized to 2 nM using the SequelPrep Normalization Plate Kit, pooled, and then measured using High Sensitivity Qubit Reagents (Life Technologies, Carlsbad, CA, USA). The library pools were denatured with 0.1N NaOH, serially diluted to 8pM with HT1, spiked with 10% 12.5pM PhiX174 bacteriophage DNA, and then sequenced using a 600-cycle MiSeq Reagent Kit v3 (Illumina Inc., San Diego, CA, USA) in four sequencing runs, two with 96 samples, one with 86 samples, and one with 87 samples.

### 16S rRNA Gene Sequence Data Processing and Analysis

Raw paired-end reads output by the MiSeq platform were merged into consensus fragments by FLASH (Magoc and Salzberg, 2011) and subsequently filtered for quality (max error rate 1%) and length (minimum 200bp) using Trimmomatic and QIIME (Caporaso et al., 2010; Kuczynski et al., 2011; Bolger et al., 2014). Spurious hits to the PhiX control genome were identified using BLASTn and removed. Passing sequences were trimmed of primers and evaluated for chimeras with UCLUST (*de novo* mode) (Edgar et al., 2011). Chloroplast contaminants were detected and filtered using the RDP classifier with a confidence threshold of 80% (Wang et al., 2007). Sequences were further screened for unknown contaminant using a sensitive BLASTn search against the GreenGenes database (McDonald et al., 2012). To normalize across samples, the 16S rRNA gene sequence profiles were subsampled to an even level of coverage prior to downstream comparisons. High-quality 16S rRNA gene sequences were assigned to a taxonomic lineage using Resphera Insight (Baltimore, MD, USA; www.respherabio.com). Mock community analyses compared Resphera Insight assignments to default UCLUST-Ref and RDP pipelines implemented within QIIME (Caporaso et al., 2010; Kuczynski et al., 2011).

Two Illumina MiSeq runs multiplexed with 96 samples produced a total of 9,631,743 and 6,949,843 quality filtered 16S rRNA gene sequences with average (average ± SD) read lengths of 412±19 and 378±27, respectively. Eighty-seven multiplexed samples in a third Illumina MiSeq run generated 8,875,924 quality-filtered sequences with an average read length of 409±24. A fourth MiSeq run of 86 samples yielded 7,631,971 quality filtered reads with aver age read lengths of 399±24. Raw 16S amplicon sequences reported in this paper have been deposited in the NCBI SRA repository (BioProject accession no. PRJNA343808).

### Resphera Insight Validation

To perform an external validation of the species level accuracy of Resphera Insight, we utilized 110 whole-genome shotgun datasets from the GenomeTrakr Project (NCBI Project ID PRJNA183844) designated as novel *S. enterica* isolates. Raw paired-end sequences were filtered for quality and length, followed by merging of overlapping sequences using FLASH (Magoc and Salzberg, 2011). Merged reads were then screened for 16S rRNA fragments using Bowtie2 (Langmead and Salzberg, 2012) against a broad database of 16S rRNA gene sequences, with additional BLAST-based filtering to confirm location specific query matches to a reference *S*. *enterica* 16S rRNA gene (*Salmonella enterica* ssp. *enterica* serovar Typhimurium strain LT2; NCBI accession NR_074910.1).

Passing sequences were submitted to Resphera Insight (Baltimore, MD, USA; www.respherabio.com) for high-resolution taxonomic identification. We also compared performance of Insight to the RDP classifier and UCLUST reference algorithms implemented within QIIME (Kuczynski et al., 2012; Navas-Molina et al., 2013). Our primary measure of performance was the *Diagnostic True Positive rate* (DTP), defined as the percentage of reads with an unambiguous assignment to *S. enterica*, and we evaluated differences in accuracy associated with changes in read length and gene position.

### Statistical Analyses

*Z* tests were performed to compare the recovery rates of *Salmonella* SAFE isolates inoculated into TT, TT_A_, and TT_B_. *Z* tests were also used to compare the efficacy of Lactose, mBPW, TSB, and UP non-selective enrichments at 5, 6, and 24 h for recovery of *Salmonella* from selective RV, TT_A_, TT_B_, and TT and to compare the efficacy of the selective enrichment broths. For the ladder comparisons the p values were adjusted for multiple comparisons, using the Benjamini and Hochberg method (R software v3.2) after continuity corrections were implemented (SAS 9.4).

## Results

### *Salmonella* Recovery from TT, TT_A_, TT_B_, and RV Selective Enrichment Broths

Since a reduced non-selective pre-enrichment will likely have fewer metabolically active cells of *Salmonella*, as compared to a 24 h pre-enrichment, we first evaluated the sensitivity of standard and modified RV and TT selective enrichment broths at a low inoculum level to mimic these conditions. To do this we directly inoculated RV and TT broths with 10^3^ CFU/mL of each of the 101 SAFE isolates. We also directly inoculated TT and RV broths with a high inoculum of 10^5^ CFU/mL as control samples. Among the selective broths inoculated with 10^3^ CFU/mL *Salmonella*, the TT_B_ and TT_A_ selective broths had significantly higher recovery rates of 92 and 88% (*P* ≤ 0.001) compared to the FDA BAM TT formulation (17%) (**Table 2**; Supplementary Table S1). The Outbreak cluster set had the lowest recovery from the BAM TT broth of 4% (**Table 2**; Supplementary Table S1). Furthermore, the Outbreak cluster and Food sets of *Salmonella* SAFE strains were recovered at 100 and 90% in TT_B_, respectively, both significantly higher than the FDA BAM TT formulations (**Table 2**; Supplementary Table S1). Interestingly, the recovery of *Salmonella* from BAM RV (58%) was also lower than TT_A_ (88%) and TT_B_ (92%) for the entire SAFE collection (**Table 2**; Supplementary Table S1).

**Table 2:** Percent recovery of *Salmonella* from selective enrichment broths.

Inoculum levels	10^3^ CFU/mL				10^5^ CFU/mL	
	RV (%)	TT (%)	TT_B_^a^ (%)	TT_A_^a^ (%)	RV (%)	TT (%)
Total SAFE collection (*n* = 101)	58	17	92^∗∗^	88^∗∗^	97	75
Subspecies Set (*n* = 55)	60	18	89^∗∗^	85^∗∗^	95	75
Outbreak Cluster Set (*n* = 26)	50	4	100^∗∗^	100^∗∗^	100	73
Food Set (*n* = 20)	65	30	90^∗∗^	80^∗^	100	80

*^a^Z test of significant difference (p < 0.001) between TT_A_ or TT_B_ and TT*

*^∗∗^p < 0.001*

*^∗^p = 0.004*

We inoculated a second set of BAM RV and TT broths with the SAFE *Salmonella* collection at 10^5^ CFU/mL and were able to recover 97 and 75% of the isolates, respectively, indicating that for some of the SAFE panel strains, the BAM selective broths require more than10^5^ CFU/mL in the food non-selective pre-enrichment for *Salmonella* recovery (**Table 2**; Supplementary Table S1). The Subspecies set of *Salmonella* isolates had the lowest recovery levels in the 10^5^ CFU/mL BAM RV (95%) and TT (75%, **Table 2**). The Outbreak cluster and Food sets of SAFE strains were recovered in 100% of the BAM RV, and in 73 and 80% in BAM TT broths, respectively, at this inoculum level (**Table 2**).

In summary, we determined that TT_A_ and TT_B_ broths significantly improved the recovery of *Salmonella* (inoculated at 10^3^ CFU/mL) and yielded positive results in 88 and 92%, respectively, of the SAFE isolates tested, as compared to the BAM TT and RV recovery rates of 17 and 58%, respectively (**Table 2**).

### Comparison of Lactose Broth to mBPW with TT, TT_A_, TT_B_, and RV Selective Broths

Before testing our reduced non-selective pre-enrichment protocol, we first tested two non-selective pre-enrichment broths (Lactose and mBPW), with three TT selective broth formulations (TT, TT_A_, TT_B_) and RV, for the recovery of *Salmonella* from cilantro, to determine the efficacy of these different media formulations on food samples. For these experiments, cilantro samples were inoculated with *Salmonella* at ∼28 CFU/25 g, and aged at 4°C prior to the 24-h non-selective pre-enrichment. The 24-h non-selective pre-enrichments were inoculated into the four selective RV and TT broths, and then plated on XLT4 for *Salmonella* isolation and confirmation (**Figure 1**). The 24-h Lactose broth pre-enrichments (*n* = 13) had recovery rates of 77% (10/13), 69% (9/13), 92% (12/13), and 92% (12/13) from the RV, TT, TT_A_, and TT_B_ selective enrichments, respectively (**Table 3**). In comparison, selective enrichment broths inoculated with 24-h mBPW non-selective pre-enrichments (*n* = 33) resulted in 100% recovery rates in all four of the selective enrichment broths, suggesting that mBPW improved resuscitation of *Salmonella* in cilantro (**Table 3**). Enumeration of three Lactose and two mBPW 24-h non-selective pre-enrichments using the MPN method revealed *Salmonella* levels of 0.00 in Lactose broth and 7.44 ± 1.15 log MPN g^-1^ in mBPW, corroborating the lower recovery rates from Lactose broth in 24-h non-selective pre-enrichments (**Table 4**).

**Table 3:** Percent recovery of *Salmonella* from cilantro non-selective pre-enrichments.

	RV (%)			TT (%)			TT_A_ (%)			TT_B_ (%)		
	5^a^	6^a^	24^a^	5^a^	6^a^	24^a^	5^a^	6^a^	24^a^	5^a^	6^a^	24^a^
**Lactose (*n* = 13)**	15	38	77	0	15	69	8	46	92	46	69	92
**mBPW (*n* = 33)**	3	9	100	0	3	100	45^∗∗^	85^∗∗^	100	52^∗∗^	97^∗∗^	100
**UP (*n* = 5)**	20	40	100	ND^b^	ND	ND	ND	ND	ND	100	100	100
**TSB (*n* = 5)**	40	40	100	ND	ND	ND	ND	ND	ND	100	100	100

*^a^Hours*

*^∗∗^Z tests of significant difference (p < 0.001) between TT_A_ or TT_B_ and corresponding (same time) RV and TT*

*using mBPW as non-selective broth*

*^b^Not Determined*

### Recovery of *Salmonella* 1 day Earlier in TT_B_

To optimize a shortened enrichment time for *Salmonella*, the four selective broths (RV, TT, TT_A_, and TT_B_) were tested in parallel with mBPW and Lactose non-selective pre-enrichments incubated for 5 and 6-h, instead of the 24-h time period recommended in the BAM (**Figure 1**). *Salmonella* was recovered one day earlier on XLT4 from 97% (32/33) of cilantro samples when TT_B_ broths were inoculated with 6-h mBPW pre-enrichments, which were found to harbor 2.00 ± 0.42 MPN g^-1^ *Salmonella* (**Tables 3** and **4**). In contrast, *Salmonella* recovery was only 69% (9/15) when TT_B_ was inoculated with a 6-h Lactose pre-enrichment, which had a correspondingly lower level of *Salmonella* contamination, 0.37 ± 0.32 MPN g^-1^ (**Tables 3** and **4**). In the other selective broths inoculated with 6-h mBPW and Lactose pre-enrichments, recovery, respectively, decreased to 85% (28/33) and 46% (6/13) in TT_A_, 3% (1/33) and 15% (2/15) in TT, and 9% (3/33) and 38% (5/13) in RV (**Table 3**). Recovery of typical-*Salmonella* colonies on XLT4 was possible but not consistent using 5-h resuscitated mBPW and Lactose broth samples, ranging from 0 to 52% depending on the selective broth used (**Table 3**). It is notable that the XLT4 plates originating from TT_B_ broth inoculated with 5 and 6-h non-selective pre-enrichments had little to no background flora, whereas those from 24-h non-selective pre-enrichments were consistently mixed cultures.

**Table 4:** Average log MPN g^-1^ *Salmonella* recovered from cilantro non-selective pre-enrichments.

	5-h (mean ± SD)	6-h (mean ± SD)	24-h (mean ± SD)
**mBPW^a^**	1.47 ± 0.16	2.00 ± 0.42	7.44 ± 1.15
**Lactose^b^**	0.37 ± 0.32	0.37 ± 0.32	0.00
**TSB^c^**	1.36 ± 1.14	2.16 ± 0.28	8.10 ± 1.58
**UP^c^**	1.79 ± 0.24	2.10 ± 0.74	8.74 ± 1.54


*^a^Four experiments for 6 and 24-h, two experiments for 5-h*

*^b^Three experiments*

*^c^Two experiments*

Two additional non-selective pre-enrichment broths, TSB and UP, were tested for their ability of to recover *Salmonella* one day earlier from selective enrichments. For these studies RV and TT_B_ were inoculated since the data support improved recovery of *Salmonella*. Similar to the mBPW results, TT_B_ resulted in higher *Salmonella* recovery compared to RV when plated on XLT4. Both UP and TSB broths resulted in recoveries of 100% (5/5) in TT_B_ and 40% (2/5) in RV using 6-h pre-enrichments that had 2.10 ± 0.52 log MPN g^-1^ (UP) and 2.16 ± 0.20 log MPN g^-1^ (TSB) *Salmonella*, respectively (**Tables 3** and **4**). Recovery of *Salmonella* was also 100% when either 5-h UP (*n* = 5) or TSB (*n* = 5) non-selective pre-enrichments containing 1.79 ± 0.17 and 1.36 ± 0.81 log MPN g^-1^ *Salmonella*, respectively, were inoculated into TT_B_ (**Tables 3** and **4**). However, recovery decreased to 20% (1/5, UP) and 40% (2/5, TSB) when the same non-selective pre-enrichments were inoculated into RV broth (**Table 3**). *Salmonella* was recovered from RV and TT_B_ control samples inoculated with 24-h UP and TSB non-selective pre-enrichments 100% of the time, and similar to the mBPW 24-h non-selective pre-enrichments, the log MPN g^-1^ *Salmonella* reached 8.74 ± 1.09 in UP samples and 8.10 ± 1.12 in TSB (**Tables 3** and **4**).

A final set of experiments with raw chicken thighs (*n* = 4), liquid whole eggs (*n* = 3), and peanut butter (*n* = 3) confirmed early detection of *Salmonella* in these foods. TT_B_ broths inoculated with 6-h non-selective pre-enrichments of chicken, eggs, or peanut butter were positive for *Salmonella* in all three foods. Additionally, using 5-h pre-enrichments, 100% of chicken and peanut butter, and 67% (2/3) of egg samples were *Salmonella*-positive. The recovery of *Salmonella* from RV broth inoculated with 6-h non-selective pre-enrichments was 100% in chicken thighs, 33% (1/3) in eggs, and 67% (2/3) in peanut butter. All 24-h chicken, egg, and peanut butter non-selective pre-enrichments resulted in positive RV selective enrichments.

In summary we significantly improved *Salmonella* recovery from cilantro by reducing the length of non-selective pre-enrichment in mBPW from 24 to 6-h, and lowering the selective strength of the BAM TT broth by removing brilliant green dye and reducing the concentration of I_2_–KI. These changes to the FDA BAM method resulted in the recovery of *Salmonella* one day early. Additionally, although a larger sample population is required for confirmation, our preliminary results with raw chicken thighs, liquid whole eggs, and peanut butter support that *Salmonella* recovery can be improved in these commodities. Taken together, our data from 6-h non-selective pre-enrichments of cilantro (excluding lactose replicates), chicken thighs, liquid whole eggs, and peanut butter enabled the recovery of *Salmonella* 1 day earlier than the BAM method in 98% (52/53) of the samples using TT_B_ and 26% (14/53) using RV supporting an overall improvement. In contrast, the FDA BAM TT broth resulted in very low recoveries of *Salmonella* from 6-h cilantro non-selective mBPW (3%) and Lactose (15%) pre-enrichments (**Table 3**).

We also tested cilantro samples in TSB and UP non-selective broths incubated for 5 and 6-h and found that *Salmonella* recovery was 100% in TT_B_ broth which is higher than the recovery from RV. However, with the small sample sizes (five samples for each broth) tested we did not have the statistical power to show a significant improvement with TT_B_ broth (**Table 3**).

### Resphera Insight Diagnostic True Positive Rates for *Salmonella*

Among species within the family *Enterobacteriaceae*, there can be high levels of similarity in 16S rRNA gene sequences, and as a result, many bioinformatics tools maintain poor sensitivity to detect *S. enterica* at the species level. Therefore, we employed the Resphera Insight algorithm for high-resolution taxonomic assignment, which we validated for accuracy on 110 novel isolates of *S. enterica* from the GenomeTrakr Project. Overall, between 16S rRNA gene positions 27 and 534, the V1 to V3 region sequenced in this study, Resphera Insight achieves diagnostic true positive rates up to 99.8% for *S*. *enterica* with improved accuracy associated with increased read length. In contrast, RDP and UCLUST were unable to achieve a DTP above 0.1% in the V1 to V3 regions (**Supplementary Figure S1**). It is notable that RDP and UCLUST had higher DTP at the 3′ end of the 16S rRNA gene at positions 800 to 900, and the highest DTP percentages with these algorithms were observed with smaller 16S rRNA gene fragments (**Supplementary Figure S1**).

### 16S rRNA Gene Amplicon Sequencing

**FIGURE 2:**
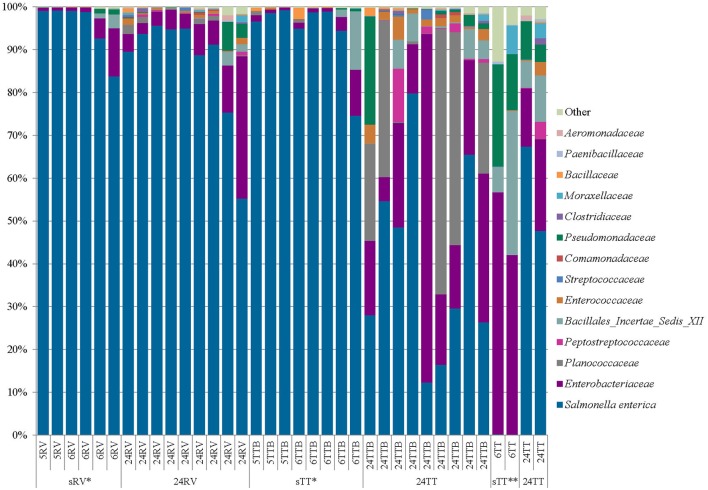
**Proportional abundances of bacteria in Tetrathionate (TT) and Rappaport–Vassiliadis (RV) selective enrichments**. Proportional abundances of *Salmonella* and other family members from TT and RV selective enrichments were estimated using 16S rRNA gene sequencing. sRV and sTT (5 and 6-h RV and TT), 24 RV and 24TT (24-h RV and TT), ^∗^ significant increase in sRV and sTT_B_ verses 24RV or 24TT_B_, ^∗∗^ significant decrease in sTT compared to 24 TT, and ^∗∗∗^ significant increase in 24RV versus 24TT_B_.

We employed 16S rRNA gene sequencing to characterize the microbial communities in selective RV, TT, and TT_B_ broth samples originating from 5, 6, and 24-h cilantro non-selective pre-enrichments (**Figures 1** and **2**). The samples sequenced included RV inoculated with 5, and 6-h non-selective pre-enrichments (sRV, *n* = 6), or 24-h non-selective pre-enrichments (24RV, *n* = 9); TT_B_ inoculated with 5, and 6-h non-selective pre-enrichments (sTT_B_, *n* = 8) or 24-h non-selective pre-enrichments (24TT_B_, *n* = 9); and TT inoculated with 6-h non-selective pre-enrichments (sTT, *n* = 2), or 24-h non-selective pre-enrichments (24TT, *n* = 2) (**Figures 1** and **2**).

Average *S*. *enterica* proportional abundances were 92% for sRV, 87% for 24RV, 92% for sTT_B_, 40% for 24TT_B_, 0.07% for sTT, and 58% for 24TT (**Figure 2**). Although all RV enrichments were similar in appearance when streaked on XLT4 plates, a multivariate linear regression analysis of *Salmonella* proportional abundances showed that sRV significantly increased *Salmonella* proportional abundances relative to 24RV (*P* = 1.58e-04) (**Figure 2**). *Salmonella* proportional abundances were also significantly higher in sTT_B_ compared to 24TT_B_ (*P* = 2.40e-05), supporting the observed reduction in background flora on XLT4 plates derived from the sTT_B_ enrichments (**Figure 2**, data not shown). Not surprisingly, the *Salmonella* abundances were significantly lower in sTT than in the 24TT selective enrichments (*P* = 2.0e-03) (**Figure 2**). The 24TT_B_ and 24TT selective enrichments had statistically similar proportional abundances of *Salmonella* while the abundances in 24RV were significantly higher than 24TT_B_ (*P* = 2.59e-05) (**Figure 2**). The proportional abundances of *Salmonella* in the sRV and sTT_B_ enrichments were statistically similar (*P* = 8.39e-01) as were those in 24RV and 24TT BAM enrichments (*P* = 1.51e-01). Finally, the proportional abundances of *Salmonella* in the sTT_B_ enrichments were significantly higher than sTT since TT broth is too harsh for a shortened non-selective pre-enrichment (**Figure 2**).

Taken together, these 16S rRNA gene sequencing data suggest that a 24-h non-selective cilantro pre-enrichment allows resident bacterial species to resuscitate, thrive, and compete with *Salmonella* during selective enrichment, whereas the shortened non-selective pre-enrichment is insufficient for resuscitation of resident bacteria and gives *Salmonella* a competitive edge. In either case, those bacteria that can metabolize the selective agents in RV and TT_B_ will grow and thrive with *Salmonella* during the selective enrichments (**Figure 2**). The 16S rRNA gene profiles of TT_B_ samples identified these competitive families as non-*Salmonella Enterobacteriaceae, Planococcaceae, Peptostreptococcaceae*, and *Bacillales Incertae Sedis XII*. Although bacteria in these families compete with *Salmonella* in TT_B_, most are Gram-positive and were not observed on XLT4 plates. However, members of the non-*Salmonella Enterobacteriaceae* family (i.e*., Citrobacter* sp. and *Enterobacter* sp.) were commonly isolated from XLT4 after enrichment in RV and TT_B_.

## Discussion

Culture based methods for pathogen detection in food are the most sensitive methods available and reducing detection times would be advantageous to food testing laboratories. Here, we were able to detect *Salmonella* one day earlier than the current FDA BAM method by reducing the non-selective pre-enrichment time in conjunction with a reformulation of TT broth. We tested this new strategy with different non-selective pre-enrichment broths and our results, comparing the efficacy of Lactose broth to mBPW, TSB, and UP, for *Salmonella* recovery from cilantro match previous studies with cantaloupes, mangos, and tomatoes which suggest that Lactose broth is not always optimal for *Salmonella* pre-enrichment (Hammack et al., 2006). In our study, even after a 24-h non-selective pre-enrichment in Lactose broth, some cilantro samples were negative for *Salmonella* but significant improvements were observed using mBPW, and although the sample sizes were too small to support significant improvements with TSA, and UP broths, all of the samples tested in these broths were positive for *Salmonella*.

An important outcome in this study was defining the enrichment dynamics of *Salmonella* amidst the complex background microbiome in cilantro. The 16S rRNA gene sequencing data revealed a significant reduction in relative abundances of competitive bacteria in sTT_B_ (8%) and sRV (8%) inoculated with 5 and 6-h non-selective pre-enrichments. The 24RV and 24TT_B_ selective enrichments had 13% and 60% proportional abundances of competitive bacteria, respectively. We also observed that TT broth did not support the recovery of *Salmonella* from 5 or 6-h non-selective pre-enrichment broths. Our results from the SAFE strain collection suggest that TT broth requires more than 10^3^ CFU/mL *Salmonella* for successful recovery, which correlates with the 5 and 6-h cilantro non-selective pre-enrichment TT results that were negative since they only contained 1.47 to 2.16 log MPN g^-1^. Additionally, the negative TT culture results were clearly supported in the 16S rRNA sequencing data where the proportional abundances of *Salmonella* in the TT broth cultures inoculated with 6-h non-selective pre-enrichments were only 0.07%. Finally, the presence of other *Enterobacteriaceae*, *Planococcaceae*, and *Pseudomonadaceae* in the TT, TT_B_, and RV enrichments suggests that bacteria in these families are resistant to the inhibitory effects in the selective broths. Further analyses are required to understand the details of the ability of these bacteria to thrive in selective enrichments.

The log MPN g^-1^ *Salmonella* levels in 6-h non-selective cilantro pre-enrichments used to inoculated selective TT_A_, TT_B_ and RV revealed that a log MPN g^-1^ of 2 to 2.16, obtained after 6-h of non-selective pre-enrichment in mBPW, UP or TSB, was sufficient to recover *Salmonella* from 97 -100% of cilantro samples. In contrast, the levels of *Salmonella* at 4-h (0.00 to 1.49 log MPN g^-1^, data not shown) and 5-h (1.36 to 1.79 log MPN g^-1^) were insufficient for consistent recovery of *Salmonella* from TT_B_ broth. There was a well-defined increase in *Salmonella* recovery from 5 to 6-h of pre-enrichment, indicating that this 6th hour of enrichment sufficiently resuscitated *Salmonella*. It is evident from the log MPN level increases from 4 to 6-h that *Salmonella* is proliferating, but for the first time, we are able to see what else is proliferating and how these other bacteria impact proportional abundances of *Salmonella* during subsequent selective enrichments. Our experiments with raw chicken thighs, peanut butter, and liquid whole eggs resulted in 100% recovery of *Salmonella* with 6-h non-selective pre-enrichments and TT_B_ suggesting that this method is effective in diverse food matrices.

The increasing use of rapid molecular methods such as qPCR and PCR for *Salmonella* detection have been highlighted in recent reviews (Park et al., 2014; Bell et al., 2016). qPCR assays require 10^2^ *Salmonella*/reaction for a positive result and as little as 30 CFU of *Salmonella* are need for consistent detection with endpoint PCR (Park et al., 2014; Bell et al., 2016). There is a consensus that a 6-h to 24-h pre-enrichment step is required for both of these methods to decrease the negative impacts on PCR and qPCR chemistry inherent to some types of food. Additional problems due to variations in competitive bacteria and inhibition due to the pre-enrichment broth itself were also noted (Park et al., 2014; Bell et al., 2016). Incorporating a 6-h selective TT_B_ enrichment simultaneous to sample preparation for qPCR and PCR would be seamless for laboratories performing these assays, and would significantly increase the overall recovery rate of *Salmonella*. Additionally, this would reduce detection time in instances where qPCR and PCR fail due to inhibition or low levels of *Salmonella*.

In conclusion, we propose that an inoculation of TT_B_ with a 6-h non-selective pre-enrichment be incubated overnight in parallel with the standard 24-h non-selective pre-enrichment in the FDA BAM method to enable the detection of *Salmonella* one day early. Our data also indicates that this will improve recovery rates of *Salmonella*.

## Author Contributions

KJ and ND designed and conducted the experiments and wrote and revised the manuscript. JW performed the Bioinformatic and statistical analyses, and assisted with manuscript preparation and revision. CG contributed to the study design, experimental analysis and manuscript preparation. DH provided funding support and overall supervision. All authors read and approved the final manuscript.

## Conflict of Interest Statement

The authors declare that the research was conducted in the absence of any commercial or financial relationships that could be construed as a potential conflict of interest.

## Supplementary Material

FIGURE S1 **Performance of Resphera Insight and other tools on novel isolates of *S. enterica***. Y-axis shows the Diagnostic True Positive Rate (DTP), i.e., the percentage of sequences assigned unambiguously to *S. enterica*. Lines show the average DTP for all reads covering each gene position for a given read length range. DTP rates over 99.5% are achieved for Resphera Insight with 300bp sequences that span the first 100bp of the 16S rRNA gene.
