# Supplementary figures and images for: Early Recovery of Salmonella from Food Using a 6-Hour Non-selective Pre-enrichment and Reformulation of Tetrathionate Broth
Source: Front Microbiol. 2016 Dec 27;7:2103. doi: 10.3389/fmicb.2016.02103 (PMC5187357; doi:10.3389/fmicb.2016.02103)

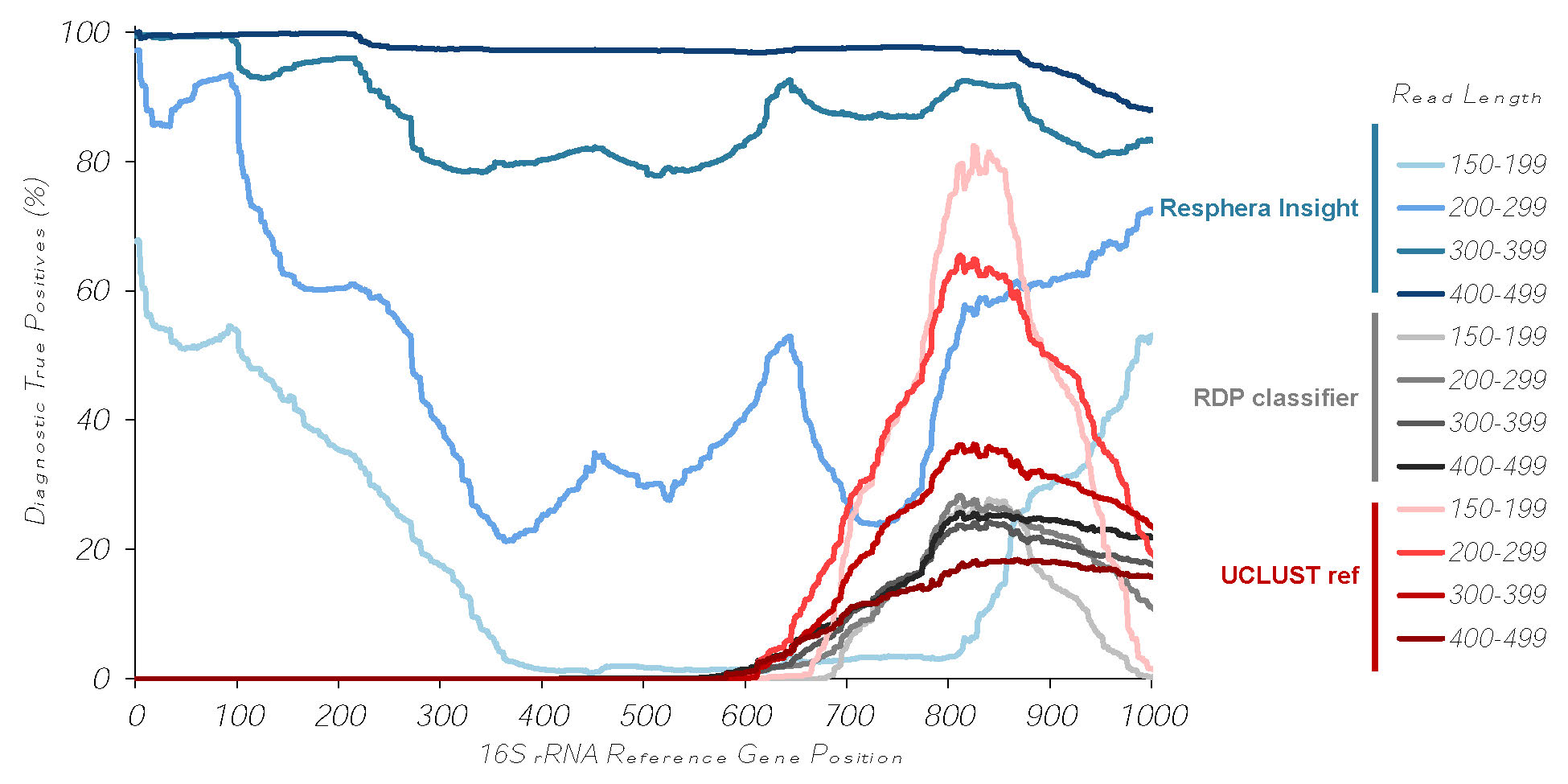

Supplement: FIGURE S1 — Performance of Resphera Insight and other tools on novel isolates of S. enterica. Y-axis shows the Diagnostic True Positive Rate (DTP), i.e., the percentage of sequences assigned unambiguously to S. enterica. Lines show the average DTP for all reads covering each gene position for a given read length range. DTP rates over 99.5% are achieved for Resphera Insight with 300bp sequences that span the first 100bp of the 16S rRNA gene. [file Image_1.JPEG]
